# Communication

**Published:** 1884-04-20

**Authors:** 


EDITORS OF THE RECORD: Sirs, I was disappointed in not meeting one or both of you at the State Medical Association. We had a very pleasant time upon the whole. Indeed, I must say that it was a decided improvement on the average meetings of the Society, and I think the next book of transactions will be the largest and best that has been issued for many years.

There are some matters that I think ought to be discussed at these meetings that are usually overlooked or evaded. Certain things are tolerated which seem to work to the interest of some, and to the detriment of others, in the profession. The profession at large is deeply interested in the matters referred to, and, if you will permit me to do so without appending my signature, I will propound a few questions which I promise will not be personal or offensive, but which look alone to the general good of the profession.

To the above note from a respectable medical friend, we reply that our pages are always open for the discussion of any question of interest not personal or offensive, and which looks to the general good of the profession. As to appending his signature, that is discretionary, though the name of the writer must be known to the editors.

